# Factors associated with disability and impact of tension-type headache: findings of the Korean headache survey

**DOI:** 10.1186/s10194-015-0524-6

**Published:** 2015-05-04

**Authors:** Byung-Su Kim, Chin-Sang Chung, Min Kyung Chu, Yun Kyung Chung, Chung-Bin Lee, Jae-Moon Kim

**Affiliations:** Department of Neurology, Bundang Jesaeng General Hospital, Daejin Medical Center, Seongnam, South Korea; Department of Neurology, Samsung Medical Center, Sungkyunkwan University School of Medicine, Seoul, South Korea; Department of Neurology, Hallym University College of Medicine, Anyang, South Korea; Department of Occupational and Environmental Medicine, Sacred Heart Hospital, Hallym University College of Medicine, Anyang, South Korea; Department of Neurology, College of Medicine, Chungnam National University, Daejeon, South Korea

**Keywords:** Disability, Epidemiology, Headache, Tension-type headache

## Abstract

**Background:**

Although mostly mild in symptom severity, tension-type headache (TTH) can cause disability. However, factors associated with disability of TTH have been rarely reported. This study sought to assess the factors associated with TTH-related disability and impact.

**Methods:**

We analyzed data form the Korean Headache Survey, a nation-wide survey regarding headache in all Korean adults aged 19–69 years. TTH-related disability was measured by surveying actual disability and Headache Impact Test-6 (HIT-6). Actual disability was defined as having one or more days of activity restriction or missed activity due to headache in the last 3 months. The HIT-6 score ≥ 50 was regarded as significant headache impact associated with TTH. We assessed factors associated with TTH-related disability and impact using logistic regression analyses adjusting for sociodemographic variables and headache characteristics.

**Results:**

Among 1507 individuals, the 1-year prevalence rate of TTH was 30.7% (n = 463), of which 4.8% reported actual disability and 21.3% had headache impact, respectively. In univariate analyses, sociodemographic variables were not associated with actual disability and headache impact, respectively. There were relationships between several headache characteristics and actual disability/headache impact. After adjustment of potential confounders, moderate headache intensity was correlated with actual disability (odds ratio [OR]: 4.41, 95% confidence interval [CI]: 1.46–13.27), while an inverse association was observed between no aggravation by routine activity and actual disability (OR: 0.32, 95% CI: 0.12–0.88). Multivariate analyses showed that ORs for headache impact were increased in those with higher headache frequency (OR: 2.54, 95% CI: 1.47–4.39 for 1–14 days/month; OR: 23.83, 95% CI: 5.46–104.03 for ≥ 15 days/month), longer headache time duration (OR: 1.84, 95% CI: 1.04–3.25 for ≥ 1 and < 4 hours; OR: 2.44 95% CI: 1.17–5.11 for ≥ 4 hours), and phonophobia (OR: 1.73, 95% CI: 1.02–2.95), whereas decreased in those with no aggravation by routine activity (OR: 0.32, 95% CI: 0.12–0.88).

**Conclusions:**

Several headache characteristics were associated with actual disability and headache impact among TTH individuals. Our findings suggest that there needs to be consideration careful of troublesome headache characteristics for TTH individuals suffering from disability and impact.

## Background

Tension-type headache (TTH) is the most prevalent headache disorder with a global prevalence of 38%, and a lifetime prevalence ranging between 30% and 78% in different studies [1-3]. TTH refers to a vague and heterogeneous headache syndrome; it is generally characterized by the absence of migrainous features as essential general diagnostic criteria, and its exact pathogenesis is still unknown [3,4]. For this reason, TTH has been given much less attention from health professionals and researchers, and there is a relative lack of epidemiological and clinical data on TTH, so far.

Recent global reports on the burden of headaches, calculated as headache days per year per person in the population multiplied by the intensity of headache, have shown that the burden of TTH was greater than that of migraine [1,5]. Similarly, the number of work days missed due to TTH was three times higher than that of migraine in previous Danish studies [6,7]. These data imply that the impact and disability caused by TTH are not always mild, contrary to its defining features.

To date, factors associated with disability of TTH have been rarely reported. Understanding the socio-demographic and headache features that were associated with TTH-related disability may be helpful to reduce the burden of its disability. Therefore, the aim of this study was to assess socio-demographic and headache characteristics associated with disability of TTH using nationwide data from the Korean Headache Survey (KHS).

## Methods

### Study population and sampling method

The KHS was a nationwide, population-based, cross-sectional study designed to investigate prevalence, demographic features, and disability of primary headache disorders in Korean adults aged 19–69 years. The details of the KHS have been previously published elsewhere [8-10]. The survey was conducted in March 2009 along with the International Conference on Harmonization’s ethical principles for medical research involving human subjects and the principles of the Declaration of Helsinki, and all study subjects received information of the study and gave informed consent [11,12]. This study was approved by the ethics review board in the Samsung Medical Center.

In terms of target area, Korea is geographically divided into 15 administrative divisions (‘do’) except Jeju-Island. In addition, each administrative division is subdivided into 60 basic administrative units (‘si,’ ‘gun’, or ‘gu’). We categorized seven ‘si’ areas (Seoul, Busan, Daegu, Incheon, Gwangju, Daejeon, and Ulsan) as ‘large cities’, other ‘si’ areas as ‘medium to small cities’, and ‘gun’ areas as ‘rural areas’ for our analysis. The estimated population of Korea in 2009 was 49,759,141 individuals according to data from the National Statistical Office, of which approximately 34,782,714 people were aged 19–69 years.

Based on the population structure, we planned to sample 1500 individuals, and a 2-stage systematic random sampling method was adopted. First, the 15 administrative divisions were designated as the primary sampling units. Proper sample numbers were assigned at each primary sampling unit in accordance with the population distribution. In the second stage, 60 representative basic administrative units were selected, in each of which we assigned a target sampling number according to age, gender, and occupation. The estimated sampling error of this study was ±2.5%, with a 95% confidence interval [10]. Of the study population who completed the survey, individuals diagnosed with TTH were included in the analysis of this study.

### Data collection

To collect study data, face-to-face interviews were conducted by trained interviewers by using a structured questionnaire. The questionnaire included socio-demographic variables, a headache profile, headache management, and headache-related disability. To minimize interest bias, we informed candidates that the survey topic was “social health issue”, rather than “headache disorder” before acceptance of survey.

### Diagnosis of tension-type headache

Based on the ICHD-2 diagnostic criteria, the diagnosis of TTH was made when subjects had experienced ten or more attacks in a lifetime, which had lasted from 30 minutes to 7 days each and were accompanied by at least two of the following four pain characteristics: mild-to-moderate intensity, bilateral location, non-pulsating quality, and no aggravation by routine physical activity. Associated symptoms could not include nausea or vomiting, but could include either photophobia or phonophobia, but not both. The validity of TTH diagnoses was further assessed by comparing the diagnoses made at the initial interview with that made by neurologists in an additional telephone interview. At the initial interview, all study participants were asked whether they would agree to have an additional telephone interview with a neurologist. An additional telephone interview was conducted with participants who agreed within 2 weeks of the initial face-to-face interview. Finally, TTH diagnosis was validated with 86.2% sensitivity and 75.5% specificity [8,10].

### Assessment of headache-related disability

During the structured interviews, information on headache-related disability was collected using the following questions: “Did you miss activities at work, school or house chores as a result of headache in the last 3 months?”, “Did you experience activity restriction at work, school or house chores as a result of headache in the last 3 months?”, and “If you experienced activity restriction or missed activity at work, school or house chores as a result of headache, How many days did you experience activity restriction or missed activity days at work, school or house chores during the previous 3 months?” In this study, an individual with headache-related disability was defined as one who had one or more days of activity restriction or missed activities (at work, school, or house chores) in the last 3 months.

To assess the headache impact on the individual’s quality of life, the KHS included the 6-item Headache Impact Test (HIT-6) [9,13,14]. Significant headache impact on the quality of life due to TTH was defined as the HIT-6 score ≥ 50, whereas individuals with the HIT-6 score ≤ 49 were considered to have little/no headache impact.

### Statistical analysis

Data of descriptive statistics were presented as means ± standard deviation or numbers (percentages). We assessed factors associated with TTH-related disability and with headache impact, respectively, through univariate and multivariate analyses adjusting for socio-demographic variables and headache characteristics. Subjects with TTH were dichotomously divided into 2 groups according to the definition of headache-related disability: no disability group versus disability group. To determine the factors associated with headache impact, subjects were classified into 2 groups as follows: little/no headache impact group versus headache impact group. Univariate logistic regression analyses were conducted to calculate the odds ratio (OR) and 95% confidence interval (CI) of disability in relation to socio-demographic variables and headache characteristics. Multivariate logistic regression analysis was performed for significantly associated (*P* < 0.05) variables from the results of univariate analyses to assess an independent predictor for disability and headache impact, respectively. The statistical analysis of the data was carried out using SPSS 18.0 (SPSS Inc., Chicago, IL. USA). All reported *P*-values were two tailed, and those < 0.05 were considered statistically significant.

## Results

### Survey and study population

Seventy six interviewers approached 4054 individuals, of which 1699 accepted the survey (rejection rate: 58.1%). Eventually, 1507 subjects completed the survey, since 191 individuals suspended the interview (cooperation rate: 37.2%). The cooperation rates did not significantly differ between the interviewers. Age, gender, size of residential area, and educational level distributions of the study sample did not show significant difference from those in the total population of Korea (Table 1). Among 1507 survey participants, 463 were diagnosed with TTH, and the 1-year prevalence rate of TTH was 30.7% (95% CI: 28.5–33.1). Of those individuals with TTH, 22 (4.8%) reported actual disability in the last 3 months: activity restriction (n = 10) and missed activities (n = 12), and 99 (21.4%) had significant headache impact (the HIT-6 score ≥ 50).

**Table 1:** **Socio-demographic distribution of 1,507 survey participants, of the total Korean population, and cases diagnosed as tension-type headache**

	**Survey Participants, n (%)**	**Total Korean population, n (%)**		**Tension-type headache**	
			***P***	**n**	**% (95% CI)**
Gender					
Men	745 (49.4^a^)	17,584,365 (49.6)	0.978^b^	243	32.5 (29.1-35.9)
Women	762 (50.6^a^)	17,198,350 (50.4)		220	29.1 (25.9-32.3)
Age (years)					
19-29	241 (22.8^a^)	7,717,947 (22.2)	0.99^b^	69	27.8 (23.1-32.6)
30-39	340 (23.5^a^)	8,349,487 (24.0)		107	31.8 (26.9-36.7)
40-49	418 (23.0^a^)	8,613,110 (24.8)		122	29.7 (24.9-34.6)
50-59	324 (19.8^a^)	6,167,505 (17.7)		117	36.5 (31.0-42.0)
60-69	184 (10.8^a^)	3,934,666 (11.3)		48	26.6 (19.8-33.5)
Size of residential area					
Large city	704 (46.7^a^)	16,776,771 (48.2)	0.89^b^	222	31.5 (28.1-34.9)
Medium-to-small city	658 (43.7^a^)	15,164,345 (43.6)		209	31.8 (28.3-35.4)
Rural area	145 (9.6^a^)	2,841,599 (8.2)		32	22.3 (15.3-29.2)
Educational level					
Middle school or less	240 (15.9^a^)	6,291,149 (19.0)	0.84^b^	79	33.8 (27.6-40.1)
High school	712 (47.2^a^)	14,530,056 (43.8)		241	34.3 (30.6-37.9)
College or more	555 (36.8a)	12,331,670 (37.2)		143	25.9 (22.5-29.4)
Total	1,507 (100.0^a^)	34,782,715 (100.0)		463	30.7 (28.5-33.1)

^a^Age- and gender-adjusted prevalence.

^b^Compared gender, age group, size of residential area, and educational level distributions between the sample of the present study and total population of Korea.

Compared tension-type headache prevalence among ^c^gender, ^d^age groups, ^e^size of residential areas and ^f^educational levels.

### Univariate and multivariate analyses for disability

Descriptive statistics on the socio-demographic and headache characteristics of the study subjects by actual disability are summarized in Table 2. In univariate analyses (Table 2), demographic variables (age, female, and BMI) did not significantly differ between individuals with and without disability. Among variables of sociodemographic status, higher education level (only college or more education), compared to lower education level (high school or less) as the reference, was marginally associated with disability, but this did not reach statistical significance (OR: 2.34, 95% CI: 0.99–5.53). In univariate analyses for headache characteristics, headache time duration ≥ 4 hours (reference: < 1 hour) was significantly associated with disability (OR: 3.73, 95% CI: 1.21–11.55). Using a category of mild headache intensity as the reference, categories of moderate and severe headache intensity were associated with disability (OR: 5.42, 95% CI: 2.17–13.51 and OR: 6.48, 95% CI: 1.26–33.30, respectively). Headache characteristic of no aggravation by routine activity was inversely associated with disability (OR: 0.26, 95% CI: 0.11–0.63).

**Table 2:** **Socio-demographic variables and headache characteristics with univariate odds ratios (95% confidence interval) for disability versus no disability**

	**Tension-type headache without disability (n = 441)**	**Tension-type headache with disability (n = 22)**	**OR (95% CI)**	***P***
Age (years)	44.0 ± 12.4	41.2 ± 12.0	0.98 (0.95–1.02)	0.305
Female	210 (47.6)	10 (45.5)	0.92 (0.39–2.17)	0.843
BMI (kg/m^2^)	23.2 ± 2.9	22.7 ± 2.4	0.94 (0.80–1.10)	0.443
Residential area				
Medium to small city/rural area	232 (52.6)	9 (40.9)	reference	
Large city	209 (47.4)	13 (59.1)	1.60 (0.67–3.83)	0.288
Educational level				
High school or less	309 (70.1)	11 (50.0)	reference	
College or more	132 (29.9)	11 (50.0)	2.34 (0.99–5.53)	0.053
Household income				
<2 million won/month	125 (28.3)	5 (22.7)	reference	
2–2.9 million won/month	124 (28.1)	8 (36.4)	1.61 (0.51–5.07)	0.413
≥3 million won/month	192 (43.5)	9 (40.9)	1.17 (0.38–3.58)	0.781
Headache frequency				
<1 day/month	205 (46.5)	7 (31.8)	reference	
1–14 days/month	226 (51.2)	14 (63.6)	1.81 (0.72–4.58)	0.208
≥15 days/month	10 (2.3)	1 (4.5)	2.93 (0.33–26.15)	0.336
Headache time duration				
<1 hour	235 (53.3)	7 (31.8)	reference	
≥1 and < 4 hours	151 (34.2)	9 (40.9)	2.00 (0.73–5.49)	0.178
≥4 hours	54 (12.2)	6 (27.3)	3.73 (1.21–11.55)	0.022
Headache intensity				
Mild	350 (79.4)	9 (40.9)	reference	
Moderate	79 (17.9)	11 (50.0)	5.42 (2.17–13.51)	<0.001
Severe	12 (2.7)	2 (9.1)	6.48 (1.26–33.30)	0.018
Bilateral location	297 (67.3)	15 (68.2)	1.04 (0.41–2.60)	0.935
Non-pulsating quality	301 (68.3)	18 (81.8)	2.09 (0.70–6.30)	0.189
No aggravation by routine activity	362 (82.1)	12 (54.5)	0.26 (0.11–0.63)	0.003
Photophobia	31 (7.0)	0 (0.0)	NA	
Phonophobia	122 (27.7)	9 (40.9)	1.81 (0.76–4.34)	0.184

Data are presented as means ± standard deviation or numbers (percentages).

Abbreviations: Abbreviations: OR, odds ratio; CI, confidence interval; BMI: Body mass index; NA, not applicable.

Based on the results of the univariate analyses, multivariate analysis was conducted adjusting headache time duration, headache intensity, and no aggravation by routine activity (Table 3). Contrary to the results of univariate analyses, headache time duration ≥ 4 hours and severe headache intensity were not significantly associated with disability in the multivariate model. Headache characteristics of moderate headache intensity and no aggravation by routine activity were independently associated with disability of TTH (OR: 4.41, 95% CI: 1.46–13.27 and OR: 0.32, 95% CI: 0.12–0.88, respectively).

**Table 3:** **Multivariable-adjusted odds ratios (95% confidence interval) of disability in relation to socio-demographic variables and headache characteristics among individuals with tension-type headache**

	**Adjusted OR (95% CI)**	***P***
Headache time duration		
<1 hour	reference	
≥1 and < 4 hours	1.16 (0.39–3.43)	0.786
≥4 hours	1.79 (0.52–6.20)	0.357
Headache intensity		
Mild	reference	
Moderate	3.93 (1.45–10.66)	0.007
Severe	4.53 (0.81–25.31)	0.085
No aggravation by routine activity	0.35 (0.14–0.87)	0.024

Abbreviations: OR, odds ratio; CI, confidence interval.

### Univariate and multivariate analyses for headache impact

In Table 4, the socio-demographic and headache characteristics of subgroups with little/no headache impact and headache impact were compared using univariate analyses. The sociodemographic variables did not significantly differ between little/no headache impact and headache impact groups. In the analyses of headache characteristics, frequent headache frequency categories, compared to infrequent headache frequency (<1 day/month), were associated with headache impact (OR: 3.03, 95% CI: 1.82–5.05 for 1–14 days/month; OR: 20.89, 95% CI: 5.19–84.14 for ≥ 15 days/month). The ORs for headache impact was increased in those with longer headache time duration (OR: 2.17, 95% CI: 1.32–3.59 for ≥ 1 and < 4 hours; OR: 3.19, 95% CI: 1.68–6.04 for ≥ 4 hours). Compared to mild headache intensity, moderate/severe headache intensity categories were positively associated with headache impact (OR: 3.15, 95% CI: 1.89–5.25 for moderate intensity; OR: 5.19, 95% CI: 1.75–15.35 for severe intensity). Phonophobia increased the risk of headache impact (OR: 2.69, 95% CI: 1.69–4.28). No aggravation by routine activity was inversely associated with headache impact (OR: 0.28, 95% CI: 0.17–0.46).

**Table 4:** **Socio-demographic variables and headache characteristics with univariate odds ratios (95% confidence interval) for significant headache impact versus little/no headache impact**

	**Tension-type headache with little/no headache impact (n = 364)**	**Tension-type headache with significant headache impact (n = 99)**	**OR (95% CI)**	***P***
Age (years)	44.1 ± 12.3	42.9 ± 12.4	0.38 (0.97–1.01)	0.992
Female	197 (54.1)	46 (46.5)	0.74 (0.47–1.15)	0.177
BMI (kg/m^2^)	23.2 ± 2.8	23.0 ± 2.99	0.98 (0.90–1.06)	0.574
Residential area				
Medium to small city/rural area	190 (52.2)	51 (51.5)	reference	
Large city	174 (47.8)	48 (48.5)	1.03 (0.66–1.60)	0.904
Educational level				
High school or less	250 (68.7)	70 (70.7)	reference	
College or more	114 (31.3)	29 (29.3)	0.91 (0.56–1.48)	0.699
Household income				
<2 million won/month	102 (28.0)	28 (28.3)	reference	
2–2.9 million won/month	105 (28.8)	27 (27.3)	0.94 (0.52–1.70)	0.829
≥3 million won/month	157 (43.1)	44 (44.4)	1.02 (0.60–1.74)	0.940
Headache frequency				
<1 day/month	188 (51.6)	24 (24.2)	reference	
1–14 days/month	173 (47.5)	67 (67.7)	3.03 (1.82–5.05)	<0.001
≥15 days/month	3 (0.8)	8 (8.1)	20.89 (5.19–84.14)	<0.001
Headache time duration				
<1 hour	207 (56.9)	35 (35.4)	reference	
≥1 and < 4 hours	117 (32.1)	43 (43.4)	2.17 (1.32–3.59)	0.002
≥4 hours	39 (10.7)	21 (21.2)	3.19 (1.68–6.04)	<0.001
Headache intensity				
Mild	301 (82.7)	58 (58.6)	reference	
Moderate	56 (15.4)	34 (34.3)	3.15 (1.89–5.25)	<0.001
Severe	7 (1.9)	7 (7.1)	5.19 (1.75–15.35)	0.003
Bilateral location	239 (65.7)	73 (73.7)	1.47 (0.89–2.41)	0.130
Non-pulsating quality	251 (69.0)	68 (68.7)	0.99 (0.61–1.60)	0.959
No aggravation by routine activity	312 (85.7)	62 (62.6)	0.28 (0.17–0.46)	<0.001
Photophobia	27 (7.4)	4 (4.0)	0.53 (0.18–1.54)	0.241
Phonophobia	86 (23.6)	45 (45.5)	2.69 (1.69–4.28)	<0.001

Data are presented as means ± standard deviation or numbers (percentages).

Abbreviations: Abbreviations: OR, odds ratio; CI, confidence interval; BMI: Body mass index.

The potential covariates from univariate analyses were finally entered in multivariate analysis (Table 5). Multivariable-adjusted ORs for headache impact were increased in those with higher headache frequency (OR: 2.54, 95% CI: 1.47–4.39 for 1–14 days/month; OR: 23.83, 95% CI: 5.46–104.03 for ≥ 15 days/month), longer headache time duration (OR: 1.84, 95% CI: 1.04–3.25 for ≥ 1 and < 4 hours; OR: 2.44 95% CI: 1.17–5.11 for ≥ 4 hours), and phonophobia (OR: 1.73, 95% CI: 1.02–2.95), whereas decreased in those with no aggravation by routine activity (OR: 0.32, 95% CI: 0.12–0.88).

**Table 5:** **Multivariable-adjusted odds ratios (95% confidence interval) of significant headache impact in relation to socio-demographic variables and headache characteristics among individuals with tension-type headache**

	**Adjusted OR (95% CI)**	***P***
Headache frequency		
<1 day/month	reference	
1–14 days/month	2.54 (1.47–4.39)	0.001
≥15 days/month	23.83 (5.46–104.03)	<0.001
Headache time duration		
<1 hour	reference	
≥1 and < 4 hours	1.84 (1.04–3.25)	0.035
≥4 hours	2.44 (1.17–5.11)	0.017
Headache intensity		
Mild	reference	
Moderate	1.69 (0.94–3.05)	0.082
Severe	3.01 (0.91–10.02)	0.072
No aggravation by routine activity	0.37 (0.21–0.66)	0.001
Phonophobia	1.73 (1.02–2.95)	0.044

Abbreviations: OR, odds ratio; CI, confidence interval.

## Discussion

In this Korean population-based study, we evaluated factors associated with TTH-related disability, in terms of actual disability and headache impact, respectively. Of TTH individuals, only a small minority of them experienced actual disability due to their headache (4.8%); however, approximately one-fifth of the TTH population had significant headache impact. The results of multivariate tests revealed that actual disability and headache impact might be influenced by several characteristics rather than by sociodemographic factors. Given the defining nature of TTH, headache characteristics might often receive less attention and be underestimated in the clinical field; however, our results suggest that headache characteristics that indicate troublesome TTH (mainly, high frequency, long attacks, and aggravation by routine activity) are useful in capturing and focusing on the most disabled subgroup.

The predictive factors for headache-related disability somewhat differed between the two multivariate tests for actual disability and for headache impact, respectively. This difference could be accounted by that two dependent factors, actual disability and headache impact, might represents different dimension of headache disability, since we used the cut-off value of some headache impact category (the HIT-6 score ≥ 50) to define significant headache impact. The HIT-6 includes diverse dimensions rather than simple loss of functioning, e.g. need for relaxation by lying down, fatigue, and irritability. This may be a reason why phonophobia was significantly related with headache impact, in contrast to the lack of an association between phonophobia and actual disability.

In the present study, actual disability such as activity restriction/missed activity was not seemingly determined by headache frequency or time duration, while the risk of headache impact was higher in those with higher headache frequency or longer duration. However, in most migraine and headache studies, headache frequency reportedly increased the burden of headache-related disability, and thus, chronic TTH has been believed to be a much noteworthy cause of significant disability than episodic TTH [15-17]. This association is contradictory to our findings. To explain this discrepancy, we may assume that most TTH attacks are not enough to induce substantial disability and this association can persist, even if headache frequency increased up to ≥ 15 days /month, whereas many migraine attacks have the potential to impair daily activities, and therefore the burden of disability is more likely to increase by headache frequency for most people with migraine. In this context, our data suggest that qualitative headache features, such as moderate intensity and no aggravation by routine activity, may play a major role in the determination of headache-related disability for population with TTH.

Although the proportion of TTH-related disability was only 4.8% in our study, this figure was sufficiently comparable to that of the disability caused by migraine, in respect of total 1507 study individuals (n = 22, 1.5% for TTH and n = 24, 1.6% for migraine) [9]. Moreover, the proportion of subgroup with some or more headache impact was greater for people with TTH than for migraine sufferers in overall study population (n = 99, 6.6% for TTH and n = 52, 3.5% for migraine) [9,10]. Therefore, the impact of disability caused by TTH should not be underestimated. Nevertheless, the number and proportion of disability related to TTH in our study were smaller than those from other previous researches [6,7,18,19]. One possible account for this is that disability, such as activity restriction or being absent from work or school, is a multifactorial outcome that can be additionally affected by individual and sociocultural factors [7,20]. For instance, a threshold for being absent may be influenced by an individual’s hiring situation and socioeconomic position, and also by medical comorbidities, such as fibromyalgia and coexisting depression/anxiety [21-23]. Furthermore, different cultural viewpoint on pain perception and sick leave could be another reason for the low proportion of disability in our study population, given the fact that Korea is one of the Asian countries where many people still believe that patience is a virtue in general [24,25].

A major strength of this study was external validity, which was well presented in previous reports using the KHS data [8-10]. However, our results should be interpreted with caution because of the following limitations. First, the analyses of cross-sectional design preclude causal inference in the present study. Second, this population-based study has good representation with low a sampling error, however small sample size can limit the statistical power of subgroup analysis, especially for multivariate logistic regression analysis for disability among individuals with TTH (Table 3). Considering number of TTH individuals with disability (n = 22), sample number for multivariate logistic regression analysis seemed to be insufficient. However, we included multivariate logistic regression analysis result for better understanding of TTH-related disability. Third, unmeasured potential confounders such as psychiatric morbidities should be mentioned, because headache-related disability could be a more complicated outcome for individuals with TTH, as described above. Fourth, according to diverse definitions or measurement methods for headache-related disability, the results could vary and be inconsistent. Since there is no standardized method to evaluate disability of TTH in contrast with migraine, development of validated study methods would be warranted in the near future to facilitate further studies on this issue [26].

## Conclusions

Given the considerable medical and social cost caused by primary headache, TTH constitute a major public health concern [27,28]. In clinical practice, better understanding of potential factors leading to headache-related disability may aid in the clinical approach to patients and for drawing a better-fitted treatment strategy. In the present study, it could be concluded that TTH individuals with higher headache frequency, longer headache time duration, moderate headache intensity, or phonophobia might be at an increased risk of actual disability or headache impact, whereas no aggravation by routine activity was associated with the decreased risk of both actual disability and headache impact. Our findings and additional studies in this area might be useful stepping stones to reduce the burden caused by headache-related disability for population with TTH.

## Abbreviations

BMI: Body mass index
CI: Confidence interval
HIT-6: Headache Impact Test-6
OR: Odds ratio
TTH: Tension-type headache
